# Successful heart transplant after 10 years of left ventricular assist device support in a highly sensitized patient

**DOI:** 10.1093/jscr/rjag141

**Published:** 2026-03-12

**Authors:** Line El Oumeiri, Constantin Stefanidis, Vanda Holovska, Frédéric Vanden Eynden, Bachar El Oumeiri

**Affiliations:** Université Libre de Bruxelles, Faculté de Médecine, Route de Lennik, 808 1070 Brussels, Belgium; Department of Cardiac Surgery, Université Libre de Bruxeles, HUB-Erasme, Route de Lennik, 808 1070 Brussels, Belgium; Department of Clinical Laboratory and Immunology, Université Libre de Bruxeles, HUB-Erasme, Route de Lennik, 808 1070 Brussels, Belgium; Department of Cardiac Surgery, Université Libre de Bruxeles, HUB-Erasme, Route de Lennik, 808 1070 Brussels, Belgium; Department of Cardiac Surgery, Université Libre de Bruxeles, HUB-Erasme, Route de Lennik, 808 1070 Brussels, Belgium

**Keywords:** LVAD, HeartWare, sensitization, heart transplantation, alloimmunization

## Abstract

Long-term left ventricular assist devices (LVAD) are an effective bridge-to-transplant strategy in end-stage heart failure. Prolonged device exposure may induce humoral activation, leading to anti-human leukocyte antigen (HLA) and non-HLA alloimmunization, complicating transplant access. We report a 43-year-old woman supported by a continuous-flow HeartWare ventricular assist device (HVAD) for 10 years, with severe anti-HLA hyperimmunization. She successfully underwent heart transplantation with stable hemodynamics, satisfactory early graft function, and no acute rejection. The postoperative course required multidisciplinary care, including hemodynamic, renal, respiratory, and infectious management, and evolved favorably. This case illustrates the feasibility of heart transplantation after prolonged LVAD support, even in highly sensitized patients, and highlights the importance of individualized immunological assessment and coordinated perioperative management.

## Introduction

Heart transplantation is the gold standard treatment for end-stage heart failure in selected patients [1]. Immunosuppressive advances have reduced acute rejection and improved survival. However, the persistent shortage of donor organs prolongs waiting times and increases mortality for patients on the transplant list [2].

Mechanical circulatory support, particularly continuous-flow left ventricular assist devices (CF-LVADs), is an effective bridge-to-transplant approach. While CF-LVADs improve hemodynamics and survival, long-term support can induce humoral activation, promoting alloimmunization against HLA, and non-HLA antigens [3, 4].

Among CF-LVADs, the HeartWare HVAD has been widely used but is associated with higher rates of thromboembolic complications, including ischemic stroke and pump thrombosis, compared with newer devices such as HeartMate 3 [5, 6]. These risks contributed to its withdrawal from the market in 2021 [7]. Thus, prolonged LVAD support represents both a mechanical therapy and a significant immunologic and thromboembolic challenge.

## Case report

A 43-year-old woman with chronic severe heart failure left ventricle ejection fraction (LVEF 16%) due to left ventricular non-compaction cardiomyopathy was admitted for orthotopic heart transplantation on 9 October 2025. She had received a HeartWare HVAD in December 2015 as a bridge to transplant.

### LVAD clinical course

During 10 years of LVAD support:

- She experienced no thromboembolic events.

- No ischemic stroke, pump thrombosis, or systemic emboli occurred.

- Device performance remained stable.

This is remarkable given the known thromboembolic risk profile of the HVAD [5, 6].

### Immunological profile

Pre-transplant Luminex SPA revealed presence of anti-HLA antibodies virtual panel reactive antibody (vPRA = 100%) (Table 1).

**Table 1:** Pre-transplant immunological profile (anti-HLA antibodies, crossmatch results).

HLA typing of the patient (PCR SSO, Lifecodes SSO, Werfen)	A^*^02, ^*^33; B^*^18, ^*^44; C^*^05, ^*^07; DRB1^*^04, −; DQB1^*^03, −; DPB1^*^04, ^*^10
HLA typing of the partner (PCR SSO, Inno-Lipa HLA Update, Fujirebio)	A^*^01, −; B^*^08, ^*^44; C^*^04, ^*^07; DRB1^*^03, ^*^11; DQB1^*^02, ^*^03
Anti-HLA specificities detected by Luminex SPA (cumulatives) – specificities corresponding to a partner HLA typing in bold (Luminex SPA, Lifecodes Single Antigen Assays, Werfen)	A1**,** A3, A11, A23, A24, A25, A26, A36, A43, A66, A80, B57, B58, B76, DR7, DR8, DR9, DR10, DR11**,** DR12, DR13, DR14, DR15, DR16, DR17**,** DR18, DR51, DR52**,** DQ2**,** DQ4, DQ5, DQ6, DQ7
Positive CDC cross-match (2017) with the partner (PBL – Total Ly population, separated T and B lymphocytes)	
Donor 1 – positive CDC cross-match (2017) – (PBL – Total Ly population, separated T and B lymphocytes) DSA in bold	A3**,** B7, B15, Cw3, Cw7, DR13**,** DR15**,** DR51**,** DR52**,** DQ6
HLA antigens defined as not acceptables for the future transplantation	A1, A3, DR3, DR8, DR11, DR12, DR13, DR14, DR52, DQ6, DQ7
Donor 2 – (2025) CDC negative cross-match (PBL and spleen lymphocytes – Total Ly population, separated T and B lymphocytes) – absence of DSA	A2, B51, B60, Cw2, Cw10, DR4, DR53, DQ8, DQA-03, DP-10, DP-0401, DPA-01

In January 2017, a positive crossmatch led to cancelation of a scheduled transplant.

Although T- and B-cell crossmatches were negative, Luminex testing demonstrated significant anti-HLA class I and II IgG antibodies, reflecting severe hyperimmunization, consistent with reports that CF-LVADs may trigger alloimmunization [3, 4].

### Transplantation and early postoperative course

Heart transplantation was performed on 9 October 2025 (ischemia time: 137 min). Weaning from cardiopulmonary bypass was uneventful under low-dose dobutamine (2 μg/kg/min), and the patient was extubated the same day with early satisfactory graft function.

During the ICU stay (October 9–20):

- Dobutamine discontinued on October 16.

- Endomyocardial biopsy on October 20: no rejection (ACR 0, AMR 0).

- Mild KDIGO stage I renal dysfunction.

- Hypoventilation managed with Optiflow, Continuous Positive Airway Pressure (CPAP), and physiotherapy.

Immunosuppressive therapy included basiliximab, mycophenolate mofetil, and methylprednisolone, with anti-infective prophylaxis using valganciclovir and trimethoprim-sulfamethoxazole [8]. She was discharged on 7 November 2025.

## Discussion

The HeartWare HVAD is associated with higher thromboembolic risk compared with other CF-LVADs [5, 6], which contributed to its market withdrawal [7]. Careful anticoagulation and monitoring are therefore crucial in long-term support.

Unlike published series, our patient experienced no thromboembolic events over 10 years, highlighting excellent device durability, effective anticoagulation, and favorable patient-device interaction. This rare outcome emphasizes that with optimal management, even devices with known risk profiles can achieve extended, safe use.

Although CF-LVADs are less immunogenic than pulsatile pumps, alloimmunization remains common [3, 4]. Severe HLA and non-HLA sensitization complicated donor matching but did not preclude transplantation. This aligns with reports showing that, with individualized immunological assessment and perioperative strategies, sensitized patients can achieve comparable short- and long-term survival [8, 9]. These findings reinforce the importance of pre-transplant immunological monitoring and tailored induction therapy.

Multidisciplinary management in this case underscores the critical role of:

Rigorous immunological surveillance, Careful donor selection, Tailored induction therapy, coordinated postoperative management in achieving favorable outcomes in high-risk patients. Comprehensive multidisciplinary collaboration ensures early detection of complications, supports optimal recovery, and can turn a high-risk transplant into a successful outcome.

## Conclusion

Heart transplantation is feasible in highly sensitized patients after prolonged LVAD support. The absence of thromboembolic events over 10 years is particularly notable. Individualized immunological assessment and multidisciplinary management are essential to optimize outcomes in this complex population.
